# Failure of Pollen Attachment to the Stigma Triggers Elongation of Stigmatic Papillae in *Arabidopsis thaliana*

**DOI:** 10.3389/fpls.2020.00989

**Published:** 2020-06-30

**Authors:** Kazuma Katano, Takao Oi, Nobuhiro Suzuki

**Affiliations:** ^1^Department of Materials and Life Sciences, Faculty of Science and Technology, Sophia University, Chiyoda, Japan; ^2^Graduate School of Bioagricultural Sciences, Nagoya University, Nagoya, Japan

**Keywords:** *Arabidopsis thaliana*, stigma morphology, heat stress, scanning electron microscope, pollen, stigmatic papillae

## Abstract

Pollination is one of key determinants of yield production in important crops, such as grains and beans in which seeds are utilized as agricultural products. Thus, to fulfil food demand for growing world population, it is necessary to elucidate the mechanisms that regulate pollination, leading to increase in yield production. In this study, we compared detailed morphological characteristics of reproductive organs in *Arabidopsis thaliana* grown under control conditions or subjected to heat stress. Shorter length of anthers, filaments, and petals were observed in plants subjected to heat stress compared to those under control conditions. In contrast, heat stress resulted in enlargement of stigma *via* elongation of stigmatic papillae. Classification of stigmas based on patterns of pollen attachment indicated that pollen attachment to stigma clearly decreased under heat stress. In addition, artificial pollination experiment demonstrated that stigma shrank when pollen attached, but, continued to enlarge in the absence of pollen. Such modulation of stigma size depending on the presence or absence of pollen was observed both under control and heat stressed conditions. Taken together, these results suggest that elongation of stigmatic papillae is associated with failure of pollen attachment to the stigma, rather than heat stress. Furthermore, histochemical staining experiments suggest that Ca^2+^ derived from pollen together with O_2_^-^ might be associated with morphological alteration of stigma depending on the patterns of pollen attachment.

## Introduction

To thrive under the natural environment, sophisticated systems of pollination that are required for proper seed production had been evolved in plants. Pollination is also known as one of key determinants of the yield production in important crops, such as grains and beans in which seeds are utilized as agricultural products. Thus, to fulfill food demand for growing world population by increasing yield production, it is necessary to elucidate the mechanisms that regulate pollination.

Flowers and buds in plants are composed of various reproductive organs. Among them, stigma was shown to be composed of approximately 150 elongated epidermal cells (stigmatic papillae) (Sessions and Zambryski, 1995). Previous studies using *Brassicae* species revealed that papillae cells have cell wall with two layers consisting of a cuticle and a pectocellulosic layer (Elleman et al., 1988; Elleman et al., 1992). These features of papillae cells might be required for the adhesion of pollen to stigma (Stead et al., 1980). Moreover, stigma might also play important roles in the processes following pollen attachment, such as pollen hydration and germination, and pollen tube elongation and navigation to ovule. For instance, certain contact-mediated competence from stigma is shown to be required for the proper guidance of pollen tubes to ovules (Palanivelu and Preuss, 2006; Qin et al., 2009).

Redox signaling involving reactive oxygen species (ROS) and nitric oxide (NO) play crucial roles in the regulation of pollen-pistil interactions (Traverso et al., 2013; Serrano et al., 2015; Katano et al., 2018a). Adhesion of pollen to stigma and hydration of pollen proceed *via* crosstalk between NO derived from pollen and H_2_O_2_ derived from stigma (Traverso et al., 2013). This crosstalk between NO and H_2_O_2_ rapidly induces release of redoxin proteins (H-type thioredoxins and glutaredoxins) from pollen coat to stigmatic papillae followed by pollen hydration and attenuation of ROS accumulation in stigma (Serrano et al., 2015). In addition, Ca^2+^ signaling that functions together with ROS and NO might also play key roles in the regulation of pollen tube elongation. For instance, pollen tube elongation can be accelerated by Ca^2+^-induced ROS signal in the tip of tubes (Cardenas et al., 2006; Potocky et al., 2007; Kaya et al., 2014). Furthermore, pollen tube navigation to the proper direction also requires crosstalk between Ca^2+^ and NO. NO that accumulates in ovary functions as a negative chemoattractant to prevent improper guidance of the direction of pollen tube elongation (Prado et al., 2004). The tip of pollen tube might sense NO derived from ovary *via* Ca^2+^ signaling (Prado et al., 2008).

Reproductive organs are known to be highly sensitive to abiotic stresses. For example, a previous study demonstrated abnormal anther development, lower pollen viability, reduced filament elongation, ovule abortion and failure of flowering in Arabidopsis plants under drought (Su et al., 2013). In contrast, enhanced elongation of stigmatic papillae was observed under high humidity (Takeda et al., 2018). These studies suggest that water status in surrounding environments can alter the developmental patterns of reproductive organs, leading to decrease in seed production. Heat stress is also known to negatively affect seed production (Suzuki et al., 2013; Katano et al., 2018b). Our previous study demonstrated the high heat sensitivity in reproductive organs of Arabidopsis mutants deficient in CYCLIC NUCLEOTIDE GATED CHANNEL 2 (CNGC2), which showed increased cytosolic Ca^2+^ influx into the cytosol under heat stress (Finka et al., 2012; Katano et al., 2018b). The high heat sensitivity of these mutants might be due to accumulation of excess H_2_O_2_ *via* dysregulation of ROS homeostasis in reproductive organs (Katano et al., 2018b). In addition, CNGC16 in Arabidopsis was also shown to play important roles in the protection of pollens against heat stress *via* activation of heat shock response induced by incorporated Ca^2+^ into the cytosol (Tunc-Ozdemir et al., 2013). Based on these findings, it could be therefore expected that heat stress might disturb coordination between Ca^2+^ signals and other integrating pathways including ROS regulatory systems which regulate development of reproductive organs and pollen-pistil interaction. However, effects of heat stress on Ca^2+^, ROS, and NO signals associated with development of reproductive organs are still largely unknown. In addition, we can expect that decrease in seed production caused by heat stress might be also at least partially attributed to morphological alterations in reproductive organs (De Storme and Geelen, 2014). Although the research of heat stress response in reproductive organs have progressed from the view point of molecular biology and physiology (Katano et al., 2018a; Suzuki and Katano, 2018), only few studies have focused on morphological alterations of reproductive organs caused by heat stress (De Storme and Geelen, 2014).

In this study, to clarify the effects of heat stress on development of reproductive organs, we analyzed morphology of each reproductive organ in Arabidopsis plants grown under control conditions or subjected to heat stress. Our observations showed that heat stress induced enlargement of stigma *via* elongation of stigmatic papillae, as well as shortened length of anthers, filaments and petals, and attenuation of pollen attachment to the stigma. Further observation of pollen attachment to stigma and experiments employing artificial pollination demonstrated that the heat induced enlargement of stigma was associated with failure of pollen attachment to stigma. In addition, morphological alteration of stigma *via* pollen attachment might be caused by crosstalk between Ca^2+^ derived from pollen, and O_2_^-^ derived from stigma.

## Materials and Methods

### Reagents

Calcium Green potassium salt and DAF-2 were purchased from Funakoshi Inc. (Tokyo, Japan) and Goryo Chemical Inc. (Sapporo, Japan), respectively. Silver nano particles with <100 nm particle size was purchased from Sigma-Aldrich, Japan (Tokyo, Japan). Other reagents were purchased from FUJIFILM WAKO Pure Chemical Co. (Osaka, Japan).

### Plant Materials and Growth Conditions

*Arabidopsis thaliana* plants (cv. Columbia-0) were grown on peat pellets (jiffy-7; http://www.jiffygroup.com/) under control conditions (21°C, 16-h light cycle, 50μmols^-1^m^-1^, 60% RH) using a growth chamber (LH-241SP, NK system, Tokyo, Japan). For the artificial pollination assay, two additional accessions of *Arabidopsis* (cv. *Landsberg erecta* and *Wassilewskija*) were also used.

### Stress Treatment

To investigate the growth characteristics of *Arabidopsis thaliana* under heat stress conditions, 25-day-old plants with approximately 1 cm of inflorescence stem grown on peat pellets as described above were transferred to a growth chamber (LH-241SP, NK system, Tokyo, Japan) with the following temperature cycle as described in Katano et al., 2018b; 06:00a.m.-09:00a.m., 21°C, 09:00a.m.-10:00a.m., 25°C, 10:00a.m.-12:00p.m., 40°C, 12:00p.m.-13:00p.m., 25°C, 13:00-06:00a.m., 21°C. Plants were then grown for additional 20 days under these temperature conditions. The 16-h light period was imposed from 06:00a.m. to 10:00p.m. Control 25-day-old plants were maintained in parallel under control conditions for additional 20 days.

For other experiments, 30-day-old plants grown on peat pellets were subjected to heat stress with the same temperature cycle as described above or maintained under control conditions for 5-9 or 10-14 days. Buds and flowers were then collected from these plants and used for the experiments as described below.

For the analyses of growth characteristics, plants were exposed to heat stress for 20 days, because relatively long duration of observation is required to monitor the number of siliques. In contrast, shorter period of heat stress was applied to flowering plants for other experiments, because we needed to obtain buds and flowers in specific developmental stages (see below) within limited durations.

### Observations of Growth Characteristics

Length of inflorescence stem, and numbers of buds, flowers and siliques of the plants grown under control conditions or subjected to heat stress were scored every two days during 20-day heat period.

Each reproductive organ was observed in buds and flowers that were collected from plants grown under control conditions, or subjected to heat stress as described above using a stereoscopic light microscope (SZX12, Olympus, Japan) and a scanning electron microscope (SEM, TM3030, Hitachi, Japan). One to two petals and sepals were removed from buds and flowers in stage 11-12 and stage 13-15, respectively (Smyth et al., 1990). Buds and flowers were photographed before and after the removal of petals and sepals. Stigma area, stigma diameter, and entire flower length, style-ovary length, anther length, filament length, petal length and sepal length as indicated in **Supplementary Figure S1** were then measured on pictures using Image J, version 6. The data of these parameters were collected from 8-20 flowers. Length of 3-4 anthers and filaments was measured per flower. Distance between stigma and anthers as indicated in **Supplementary Figure S1** was also measured on the pictures using Image J, version 6. This parameter was analyzed by measuring distance between stigma and two anthers which were nearest to the stigma.

Fine structure of stigma, anthers and pollen in buds and flowers corresponding to stage 11-12 and 13-15, respectively (Smyth et al., 1990) were observed under SEM. Flowers or buds collected from plants grown under control conditions or subjected to heat stress were mounted on a stub with adhesive carbon tape, and transferred directly to specimen chamber of the SEM in low vacuum condition.

### Classification of Stigmas Based on the Patterns of Pollen Attachment

Patterns of pollen attachment to the stigma were randomly analyzed in 28-31 flowers collected from 10 plants grown under control conditions or subjected to heat stress. Flowers collected were photographed under a stereoscopic light microscope and stigmas of these flowers were classified into four types based on the patterns of pollen attachment (**Supplementary Figure S2**). Type 1 represents stigmas almost completely covered with pollen. Type 2 represents stigmas with pollen attached to approximately half area. Type 3 represents stigmas with pollen attached only to an edge of stigma. Type 4 represents stigma with no pollen. Then, proportion of each type of stigma under controlled or heat stressed conditions was calculated.

### Artificial Pollination Assay

All buds and flowers except for the one biggest bud corresponding to stage 12 were removed from 30-day-old plants grown under control conditions as described above. Anthers were then removed from this remaining bud. Twenty-four hours after the emasculation, pollen from other Columbia-0 plants grown under control conditions were attached to the stigma of this remaining bud. Five to six anthers were taken from the pollen donor plants with tweezers and rubbed on the stigmas to artificially attach the pollen. One hour after the artificial pollination, plants were subjected to moderate heat stress (35°C) for 1 h followed by recovery under control conditions for 24 and 48 h. In this experiment, moderate heat stress was applied to buds, because heat stress (40°C, 2 h) employed in the analyses of growth characteristics and morphology of reproductive organs as described above was too strong for buds without sepals and petals. Control plants were maintained in parallel under control conditions. Stigmas were photographed before artificial pollination and at 24 and 48 h following the recovery from heat application (i.e. 26 or 50 h following the artificial pollination). Diameter of stigmas was then measured on the pictures using Image J, version 6. Changes of stigma diameter were calculated by subtracting diameter before artificial pollination from that at 26 or 50 h following the artificial pollination. Stigmas without artificial pollination were also photographed at the same time points in parallel and analyzed in changes of stigma diameter. In addition, silver nano particles (<100 nm particle size) or dead pollen as mechanical controls were also attached to the stigma at the same timing as the artificial pollination described above. Dead pollen were obtained from anthers in plants subjected to severe heat stress (42°C, 16 h). A recent study demonstrated that visible degeneration of stigmatic papillae started around at 3 days after emasculation (Gao et al., 2018). Therefore, the experiments were designed so that we performed final observation of stigma at 74 h after emasculation.

### Histochemical Staining

*In situ* detection of O_2_^-^, Ca^2+^, and NO using nitro blue tetrazolium (NBT), Calcium Green solution and Diaminofluorescein-2 (DAF-2), respectively were performed as previously described (Xing et al., 2013; Iwano et al., 2014; Farnese et al., 2017). Flowers with type1 or type 4 stigma were detached from plants grown under control conditions or subjected to heat stress (10-14 days). For O_2_^-^ detection, flowers were submerged in NBT solution (1 mg ml^-1^ NBT plus 10mM NaN_3_ solution in 10mM potassium phosphate buffer pH7.8) and incubated for 40 min at room temperature. Then, flowers were boiled in 95% ethanol for 15 min to completely remove the chlorophyll and stored in 60% glycerol. Flowers were observed under a stereoscopic light microscope and photographed. For Ca^2+^ detection, a drop of 10 μM Calcium Green solution containing 0.005% Tween 20 was put on flowers. After incubation at room temperature for 1 h, fluorescent signals in flowers were detected by a fluorescence microscope (LeicaMZ10 F) using 480/40nm excitation and 510 nm emission filter (GFP2 Plus). For NO detection, flowers were submerged in 10 μM of DAF-2 solution in the dark for 20 min at room temperature. Fluorescent signals were detected by a fluorescence microscope (LeicaMZ10 F) using 480/40 nm excitation and 510 nm emission filter (GFP2 Plus). The same experiments were also performed using buds corresponding to stage 11-12 (Smyth et al., 1990).

### Alexander Staining

Pollen staining with the Alexander dye was performed as previously described (Zhou et al., 2013). Anthers were collected from buds corresponding to stage 11-12 (Smyth et al., 1990) in plants grown under control conditions or subjected to heat stress. Anthers were then fixed with a 6:3:1 (v:v:v) of ethanol: chloroform: acetic acid for 2 h. Following the fixation, anthers were put on a microscope slide and 2 or 3 drops of Alexander solution were applied. Slides were slowly over an alcohol burner in a fume hood until the stain solution is near boiling (approximately 30 s). Anthers were then photographed under a microscope.

### Statistical Analyses

Two-Way ANOVA followed by Tukey's test, or Student's *t*-test was performed using R package (CRAN: https://cran.r-project.org/).

## Results

### Increased Number of Flowers Under Heat Stress

To investigate the effects of heat stress on the development of Arabidopsis plants during reproductive stage, 25-day-old plants were grown under control conditions or subjected to heat stress for additional 20 days and analyzed in length of inflorescence stem, and numbers of buds, flowers and siliques (**Supplementary Figure S3**). Inflorescence stem of plants subjected to heat stress was significantly longer compared to that grown under control conditions at 18 and 20 days following the heat stress application (**Supplementary Figure S3A**). Numbers of buds under heat stress were comparable to that under control conditions, except for 0 to 4 days following the heat stress application (**Supplementary Figure S3B**). However, number of flowers in plants subjected to heat stress was significantly increased compared to that under control conditions at 20 days following the heat stress application (**Supplementary Figure S3C**). Because no aborted flowers and buds were observed in our study, thus, heat stress might promote early flowering. No significant differences were observed in number of siliques between control and heat stressed conditions (**Supplementary Figure S3D**). Among three different types of reproductive organs (i.e. buds, flowers and siliques), only flowers demonstrated significant increase in the number by heat stress. Thus, we further investigated effects of heat stress on development of various reproductive organs mainly in flowers.

### Enlargement of Stigma Under Heat Stress

**Figure 1:**
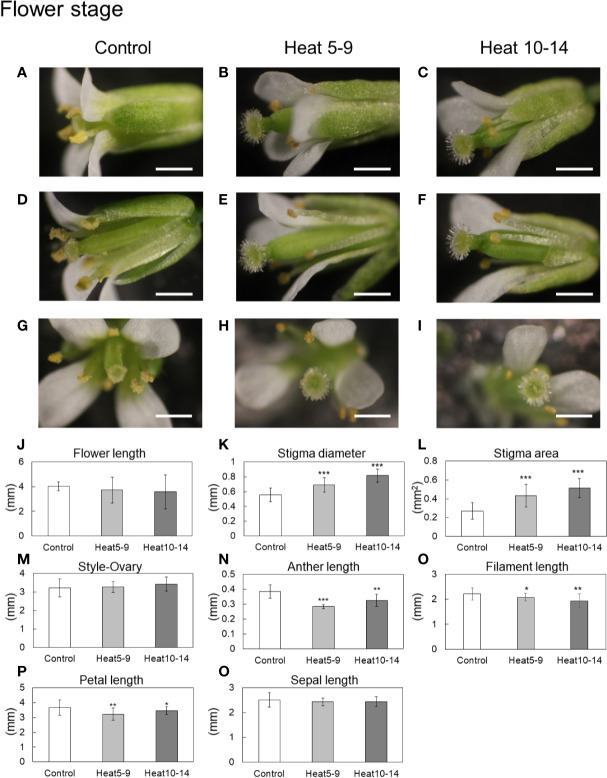
Morphological alterations of reproductive organs in wild type plants of Arabidopsis (cv. Columbia-0) by heat stress. Plants in reproductive stage were subjected to daily heat stress for 5–9 days (Heat 5–9) or 10–14 days (Heat 10–14). **(A–C)** Flowers under control or heat stressed condition. **(D–F)** Flowers after the removal of petals and sepals for the observations. **(G–I)** Top view of flowers. **(J)** Flower length. **(K)** Stigma diameter. **(L)** Stigma area. **(M)** Length between style and ovary. **(N)** Anther length. **(O)** Filament length. **(P)** Petal length. **(Q)** Sepal length. White bars in pictures indicate 1 mm. Bars in graphs indicate standard error. *, ** and ***: Student *t*-test significant at p < 0.05, p < 0.01 and p < 0.005, respectively compared to control (n=6–20).

To investigate morphological alterations of various reproductive organs caused by heat stress, we observed buds and flowers of plants grown under control conditions or subjected to heat stress under a stereoscopic light microscope, and measured the size or area of each reproductive organ (**Figure 1**; **Supplementary Figure S4**). Significantly larger area of stigma was observed in flowers subjected to heat stress compared to that under control conditions (**Figures 1A-I, K, L**). On the other hand, length of anthers and filaments, and length of petals were reduced in flowers subjected to heat stress when compared to that under control conditions (**Figures 1D-F, N–P**). In addition, no significant effects caused by heat stress were observed in length of flowers, sepals and style-ovary (**Figures 1D-F, J, M, P**). In contrast to flowers, heat stress did not clearly alter the morphology of reproductive organs including stigma diameter and area in buds (**Supplementary Figure S4**).

### Elongation of Stigmatic Papillae and Decreased Pollen Attachment to Stigma Under Heat Stress

**Figure 2:**
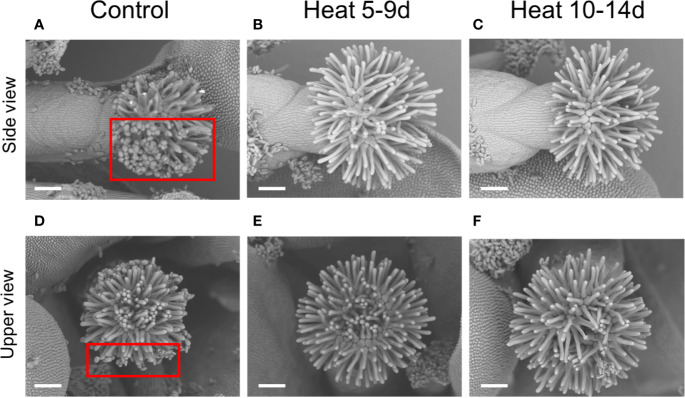
Stigmas in flowers of Arabidopsis plants (Colombia-0) photographed under a scanning electron microscope. **(A–C)** Side view of stigmas grown under control conditions **(A)** or subjected to heat stress **(B, C)**. **(D–F)** Top view of stigmas grown under control conditions or subjected to heat stress. Squares in **(A, D)** indicate the area of pollen attachment on stigma. Bars indicate 100 μm.

To further analyze morphological alterations in reproductive organs caused by heat stress, we observed buds and flowers of plants grown under control conditions or subjected to heat stress by SEM (**Figure 2**). Stigmatic papillae in flowers subjected to heat stress were clearly longer than that under control conditions (**Figures 2A–F**). Thus, enlargement of stigmatic area is due to elongation of hairy-shaped papillae (**Figure 2**). Although only few pollen attached to stigmatic papillae in flowers subjected to heat stress, attachment of many pollen were observed on stigmatic papilla which seemed to be shrunk under control conditions (**Figure 2**, indicated in rectangles). We also observed stigmas in buds by SEM (**Supplementary Figures S5A–C**). Only slight elongation of stigmatic papillae was observed in buds subjected to heat stress when compared to that under control conditions (**Supplementary Figures S5A–C**). These results indicate that enlargement of stigma due to the elongation of stigmatic papillae might be resulted from the failure of pollen attachment to stigmatic papillae caused by heat stress, especially in flowers. In contrast to stigma, structure of anthers and pollen subjected to daily heat stress for 5-9 or 10-14 days was comparable to that under control conditions both in flowers and buds (**Supplementary Figures S5D–F** and **S6**).

### Heat Stress Triggers Decrease in Pollen Attachment to Stigma

**Figure 3:**
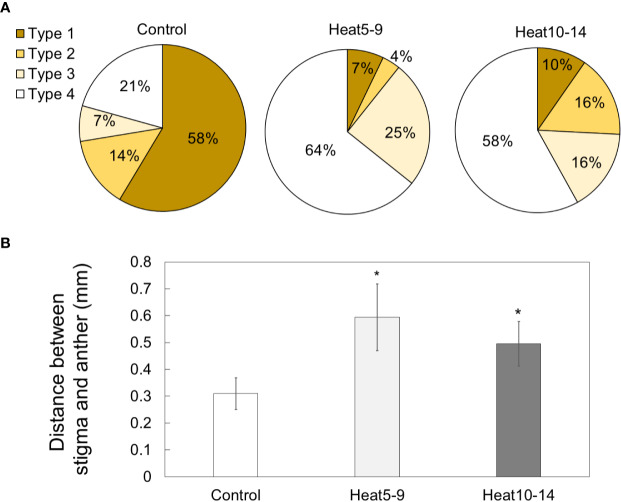
Analysis of pollen attachment patterns to the stigma and distance between anthers and stigma in flowers of Arabidopsis plants (Columbia-0). **(A)** Stigmas were classified into four types based on the pollen attachment patterns (refer to **Supplementary Figure S2**) and proportion of each type of stigma relative to total number of stigmas was calculated. **(B)** Distance between anthers and stigma grown under controlled or heat stressed conditions. Bars indicate Standard error. *, Student *t*-test significant at p < 0.05 compared to control (n = 13–21).

SEM observation indicated that pollen attachment to stigmatic papillae can be decreased by heat stress (**Figure 2**). To investigate effects of heat stress on pollen attachment to stigma, we classified stigmas of flowers grown under control conditions or subjected to heat stress into 4 different types based on the patterns of pollen attachment as indicated in **Supplementary Figure S2** (Please also see *Classification of Stigmas Based on the Patterns of Pollen Attachment* in *Materials and Methods*). Fifty-eight percent of stigmas under control conditions were classified into type 1, and 14%, 7%, and 21% of stigmas were classified into type 2, 3, and 4, respectively. On the other hand, only 7% of stigmas subjected to heat stress for 5-9 days were classified into type 1, and 4%, 25%, and 64% of stigmas were classified into type 2, 3, and 4, respectively. Similar results were also obtained in stigmas subjected to heat stress for 10-14 days; 10%, 16%, 16%, and 58% of stigmas were classified into type 1, 2, 3, and 4, respectively (**Figure 3A**). To further investigate cause of the decrease in pollen attachment to stigma under heat stress, we measured the distance between stigma and two anthers which were nearest to the stigma (**Figure 3B**). Plants subjected to heat stress for either 5-9 or 10-14 days showed significantly longer distance between stigma and anthers compared to that under control conditions. To investigate impact of heat stress on pollen, we analyzed pollen viability in buds of plants grown under control conditions or subjected to heat stress by Alexander staining (**Supplementary Figure S7**). While many viable pollen were observed in buds under control conditions, almost no pollen were viable in buds subjected to heat stress (**Supplementary Figures S7A, B**). Taken together these results indicate that heat stress negatively impacts on pollen attachment to stigma as well as pollen viability.

### Pollen Attachment Causes Shrinkage of Stigma

**Figure 4:**
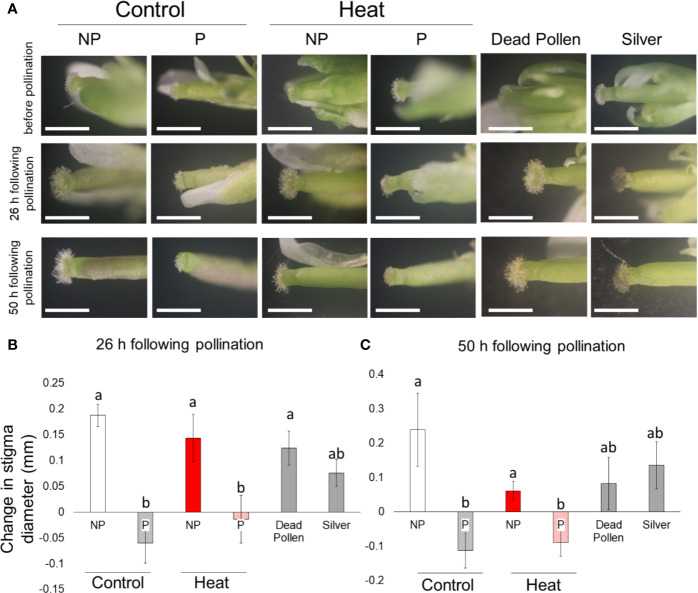
Effects of artificial pollination on stigma diameter in flowers of Arabidopsis plants (Columbia-0) under controlled or heat stressed conditions. Columbia-0 plants also used as Pollen donor plants. **(A)** Photographs of stigmas before and after artificial pollination. NP, no pollen on the stigma; P, pollen on the stigma. Dead pollen and silver nano particles also attached to the stigma as mechanical controls. Scale bars indicate 1 mm. **(B)** Change in stigma diameter at 26 h after artificial pollination and **(C)** 50 h after artificial pollination compared to that before artificial pollination under controlled or heat stressed conditions. Bars indicate Standard error. Two-way ANOVA followed by Tukey's test was performed (n=5). Values statistically different at P<0.05 are denoted with different letters (a and b).

Enlargement of stigma and decrease in pollen attachment to stigma was observed in flowers subjected to heat stress (**Figures 2** and **3A**). These findings prompted us to confirm if failure of pollen attachment to stigmatic papillae resulted in stigmatic enlargement, or if heat stress itself enhanced stigmatic enlargement independently of pollen attachment. To analyze effects of pollen attachment on size of stigma, we compared diameter of stigmas with or without artificial pollination (**Figure 4**). The similar experiment was conducted in parallel under heat stress to test if heat stress itself can cause enlargement of stigma. Enlargement of stigma without pollen was observed under control conditions (**Figures 4A–C**). On the other hand, attachment of pollen resulted in shrinkage of stigma under control conditions at 26 h and 50 h following the artificial pollination (**Figures 4A–C**). Similar results were obtained in plants subjected to heat stress. These results demonstrate that enlargement of stigma was induced by failure of pollen attachment, rather than heat stress. Significant differences were not observed between stigma with pollen and that with silver nano particles or dead pollen. However, attachment of dead pollen did not result in obvious shrinkage of stigma (**Figures 4A–C**), suggesting that attachment of viable pollen might be required for shrinkage of stigmatic papillae. Indeed, we also demonstrated that no viable pollen were observed under heat stress (**Supplementary Figure S7**). Thus, abolishment of viable pollen by heat stress might also induce enlargement of stigma. Furthermore, physical stimulus is not an inducer of shrinkage of stigma, because attachment of silver nano particles did not clearly alter the size of stigma (**Figures 4A–C**). Moreover, the same experiment was conducted using two different accessions of Arabidopsis (cv. *Landsberg erecta* and *Wassilewskija*), and similar results were obtained (**Supplementary Figures S8A, B**).

### High Accumulation of Ca^2+^ in Stigma With Rich Pollination in Flowers

**Figure 5:**
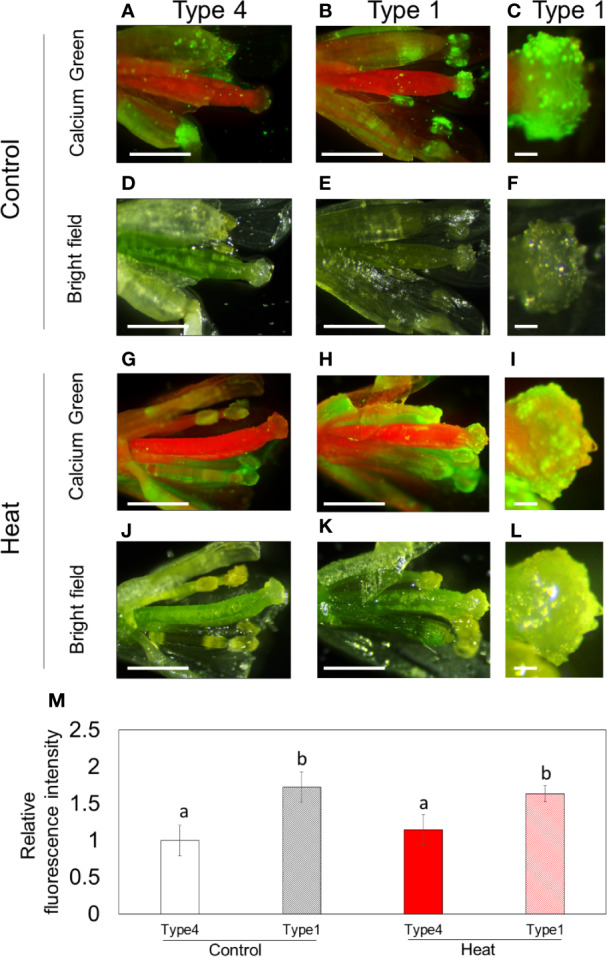
Distribution of Ca^2+^ in reproductive organs of Arabidopsis plants (Columbia-0) grown under control conditions or subjected to heat stress. **(A–L)** Calcium green staining of reproductive organs in flowers with rich (type 1) or poor (type 4) pollen attachment under control **(A–F)** or heat stressed **(G–L)** conditions. **(C, F, I, L)** Enlarged figures of stigma in **(B, E, H, K)**, respectively. **(D–F, J–L)** Bright field images. Scale bars in **(A, B, D, E, G, H, J, K)** indicate 1mm, and scale bars in **(C, F, I, L)** indicates 100 μm. **(M)** Relative fluorescence intensity of Ca^2+^ level in stigma. Bars indicate standard error. Two-way ANOVA followed by Tukey's test was performed (n=5). Values statistically different at P<0.05 are denoted with different letters (a and b).

Previous studies revealed that crosstalk between Ca^2+^, O_2_^-^, and NO signaling regulates multiple processes of reproductive development and pollen-pistil interaction (Prado et al., 2004; Traverso et al., 2013; Serrano et al., 2015; Katano et al., 2018a). Therefore, to investigate the roles of these signals in stigma development, we analyzed distribution and accumulation of Ca^2+^, O_2_^-^, and NO in flowers (**Figure 5**). In these analyses, flowers with type 1 or type 4 stigma were used (**Supplemental Figure S2**). In flowers with type 4 stigma, high accumulation of Ca^2+^ was detected in pollen, but, not in stigma under control conditions (**Figure 5A**). In contrast, in flowers with type 1 stigma, high level of Ca^2+^ accumulated in stigma as well as pollen attached to it under control conditions (**Figures 5B, C**). Similar trends were observed in flowers subjected to heat stress (**Figures 5G–I**). In addition, fluorescent intensity of Ca^2+^ in type1 stigma was significantly higher than that in type 4 stigma both under control and heat stressed conditions (**Figure 5M**). These results indicate that high accumulation of Ca^2+^ derived from pollen might be associated with shrinkage of stigmatic papillae. We also analyzed distribution of Ca^2+^ in buds under control or heat stressed conditions (**Supplementary Figure S9**). In contrast to flowers, Ca^2+^ highly accumulated in anthers, but not in stigma under both conditions (**Supplementary Figures S9A–D**).

### High Accumulation of O_2_^-^ in Whole Flowers With Pollinated Stigma, and Detection of NO in Stigma

**Figure 6:**
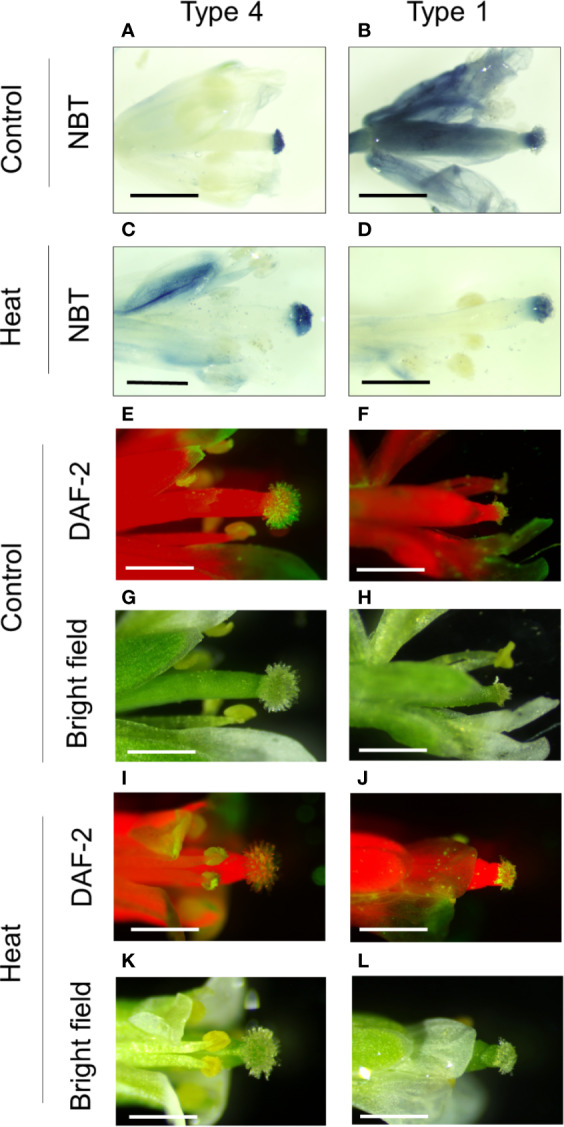
Distribution of O_2_^-^ and NO in reproductive organs of *Arabidopsis thaliana* grown under control conditions or subjected to heat stress. **(A–D)** NBT staining of reproductive organs in flowers with rich (type 1) or poor (type 4) pollination under control **(A, B)** or heat stressed **(C, D)** conditions. **(E–L)** DAF-2 staining of reproductive organs in flowers with rich (type 1) or poor (type 4) pollination under control **(E, F)** and heat stressed **(I, J)** conditions. **(G, H, K, L)** Bright field images. Bars indicate 1mm.

In contrast to Ca^2+^, in flowers with type 4 stigma, O_2_^-^ was only detected in stigma under control conditions. However, in flowers with type 1 stigma, O_2_^-^ spread to style and ovary, but not highly accumulated in stigma under control conditions (**Figures 6A, B**). Under heat stress, similar patterns of O_2_^-^ accumulation were observed in type 1 stigmas, but O_2_^-^ spread was not observed in flowers with type 1 stigma (**Figures 6C, D**). These results indicate that elongation of stigmatic papillae might be associated with O_2_^-^ accumulation. In addition, detectable level of NO was observed in stigma with either type 4 or type 1 stigma under control and heat stressed conditions, but, clear differences in fluorescent intensity were not observed among them (**Figures 6E–L**). Furthermore, we also analyzed the distribution of O_2_^-^ and NO in buds under control or heat stressed conditions. In contrast to flowers, clear accumulation of O_2_^-^ and NO was not detected in reproductive organs of buds both under control and heat stressed conditions (**Supplementary Figures S9E–J**).

These results obtained from the analyses of buds also support the importance of these signals that are associated with pollen-pistil interaction in the regulation of size of stigma, because alteration in the Ca^2+^, O^2+^ and NO distributions, as well as size of stigma caused by heat stress was clearly observed in flowers in which pollen-pistil interaction proceeds, but not in buds (**Supplementary Figures S4K, L** and **Figure S9**).

## Discussion

We discovered a novel phenomenon that pollen attachment to stigma is clearly associated with stigma shrinkage, and failure of pollen attachment to stigma by heat stress leads to elongation of stigmatic papillae. Analysis employing artificial pollination also confirmed that size of stigma is modulated depending on the status of pollen attachment, rather than heat stress (**Figure 4**). This stigmatic enlargement when insufficient pollen attach to stigma might had been evolved in plants to increase the possibility of pollination and fertilization success under fluctuating temperature. In addition, numerous studies have strongly pointed out that attenuation of pollen production in anthers is associated with the sensitivity to heat stress (De Storme and Geelen, 2014; Rieu et al., 2017). Our results indicate that morphological alteration in stigma, as well as protection of pollen production, might be also an important factor to prevent the abortion of pollination during heat stress. Furthermore, our artificial pollination assays using silver nano particles or dead pollen also indicate that attachment of living pollen, not physical stimuli might be a key for the stigma shrinkage. Enlargement of stigma without pollen was also observed even in *Ler* and *Ws* plants (**Supplementary Figure S8**). These results indicate that enlargement of stigma induced by failure of pollen attachment might be conserved process among different Arabidopsis ecotypes. Moreover, hypothesis suggested in our study might be also applied for other plant species, because molecular mechanisms underlying development of reproductive organs are conserved in various species including trees to some extent (Yoo et al., 2012; Cronk et al., 2020). A previous study demonstrated that elongation of stigmatic papillae can be also induced by high humidity (Takeda et al., 2018). In contrast to our study, enlargement of stigma induced by high humidity was not associated with pollen attachment (Takeda et al., 2018), suggesting that effects of pollen attachment on stigmatic development might be different depending on humidity. Thus, it is necessary to study effects of various environmental factors on stigmatic development.

Decrease of pollen attachment to stigma might be caused by heat-induced morphological alterations of reproductive organs. Heat stress shortened filament length, but, did not affect style-ovary length in flowers (**Figures 1O, M**). In addition, longer distance between stigma and anthers was observed in plants subjected to heat stress compared to that under control conditions (**Figure 3B**). These results indicate that decrease in pollen attachment to stigma caused by heat stress might be at least partially due to shortening of stamen which might lengthen the distance between stigma and anthers.

Several studies reported that development of gynoecium with proper timing and morphology is necessary to achieve efficient pollination followed by seed production, as it needs to receive pollen that are released from anthers in certain limited timing (Goldberg et al., 1993; Alvarez and Smyth, 2002). The *Hecate* (*Hec*), *Spatula* (*Spt*), and *Half filled* (*Haf*) genes which encode putative basic helix-loop-helix (bHLH) transcription factors are known to be the main regulators of reproductive tract as they control overall growth of stigma, style and transmitting tract (Heisler et al., 2001; Gremski et al., 2007; Crawford and Yanofsky, 2011). It should be important to address whether these transcription factors are involved in the morphological alterations of stigma observed in this study. Furthermore, mutants of *Hec*, *Spt*, and *Haf* genes showed reduced fertility (Heisler et al., 2001; Gremski et al., 2007; Crawford and Yanofsky, 2011), suggesting that proper development of reproductive organs including stigma is tightly linked to the fertility. Thus, it is also necessary to investigate how alterations of stigmatic morphology can affect fertility. To address these questions, morphology of stigma in the presence or absence of pollen as well as fertility should be investigated in mutants deficient in *Hec*, *Spt*, and *Haf* genes. Furthermore, a rent study demonstrated that programmed cell death (PCD) dependent-degeneration of stigmatic papillae can be started around at 3 days after emasculation (Gao et al., 2018). It is therefore important to investigate how pollen attachment affect PCD pathways in stigma.

**Figure 7:**
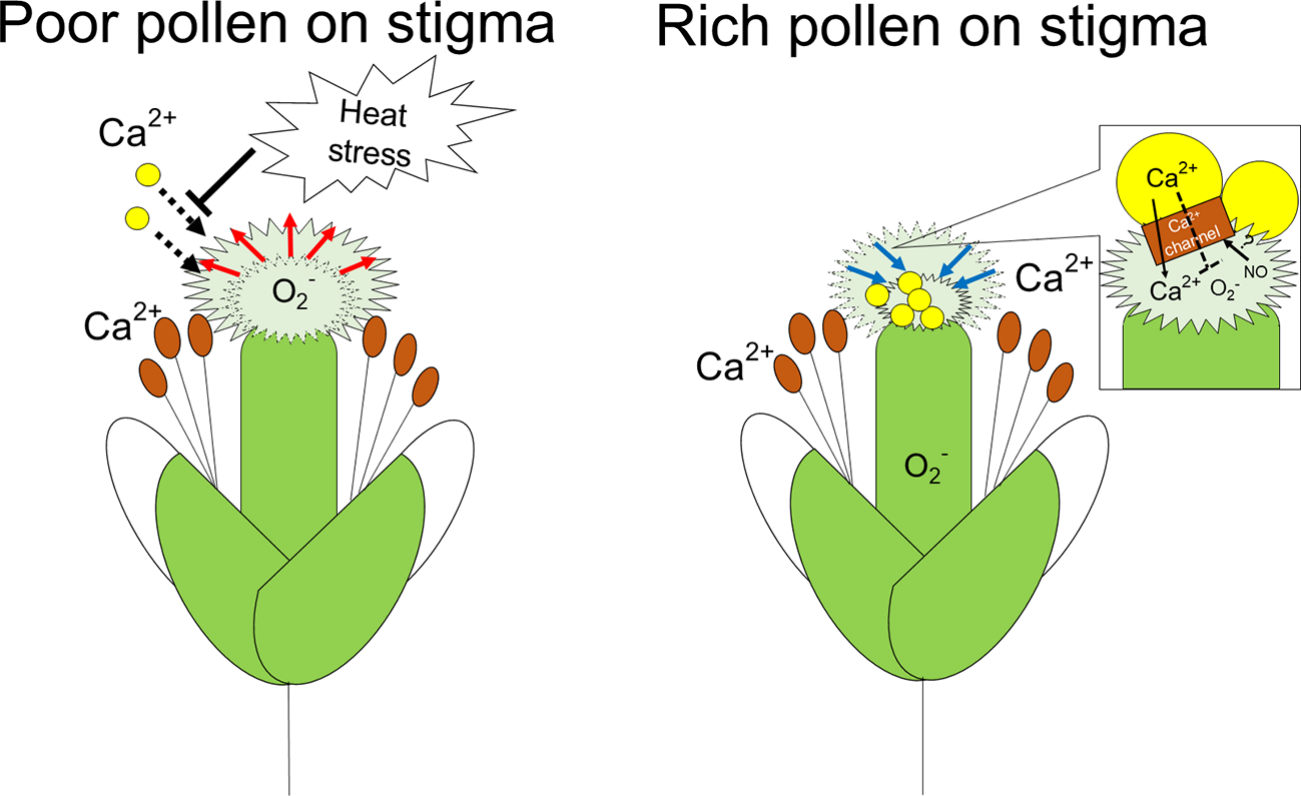
A scheme of putative mechanisms regulating development of stigma with poor or rich pollinations. When pollen attachment is poor, enlargement of stigma *via* elongation of stigmatic papillae might be activated by the functions of O_2_^-^ as a signaling molecule. In contrast, when many pollen attached to the stigma, Ca^2+^ signaling derived from pollen might function to reduce the size of stigma *via* shrinkage of papilla cells. NO might activate calcium permeable channel to assist Ca^2+^ entry into the stigma. Decrease of O_2_^-^ accumulation in stigma might be induced by Ca^2+^ signaling. Spread of O_2_^-^ distribution to style and ovary might be associated with processes of pollen-pistils interaction following the attachment of pollen to the stigma.

In this study, we revealed that distribution of Ca^2+^ and ROS in reproductive organs can be altered depending on the presence or absence of pollen on stigma. Especially, high accumulation of Ca^2+^ was clearly observed in stigmas with rich pollinations (i.e. type 1), as well as pollen (**Figure 5**). On the other hand, O_2_^-^ highly accumulated in stigma with poor pollinations (**Figure 6**). These results suggest that enlargement of stigma with poor pollinations might be induced *via* functions of O_2_^-^. In contrast, when many pollen attached, Ca^2+^ signaling derived from pollen might function to reduce the size of stigma (**Figure 7**). Several studies indicated that crosstalk between Ca^2+^ and ROS signaling plays quite important roles in the regulation of the development of reproductive organs (Katano et al., 2018a). For instance, production of mature pollen grains in anthers requires a proper timing of tapetum degradation *via* functions of RBOH-dependent ROS production that might be activated by Ca^2+^ signaling in *Arabidospsis thaliana* (Xie et al., 2014). In addition, previous studies indicated the involvement of Ca^2+^ in the regulation of tip growth of cells. Ca^2+^ gradient with high accumulation in the tip was shown to be required for growth of pollen tubes and root hairs (Bibikova et al., 1997; Cardenas et al., 2008). However, Li et al., demonstrated that high concentration of extracellular Ca^2+^ inhibits elongation of pollen tubes (Li et al., 1999). These findings suggest that level of Ca^2+^ should be strictly balanced to modulate tip growth. It is therefore necessary to further elucidate how Ca^2+^ and other integrating signals inhibit tip growth of stigmatic papillae in future studies. Furthermore, O_2_^-^ and NO were not detected in each reproductive organ in buds under controlled and heat stress conditions. Considering the only slight enlargement of stigma in buds, these results also support the importance of these signals for morphological alteration of stigmatic papillae especially in flowers.

We can expect that morphological alterations of stigma accompanied by alterations in Ca^2+^ and ROS signaling might be also required for sequential processes following pollen attachment to stigma; pollen adhesion, pollen hydration and germination, and pollen tube elongation and navigation to ovule (Traverso et al., 2013). Indeed, it was demonstrated that signals from stigma might be required for the proper guidance of pollen tubes to ovules (Palanivelu and Preuss, 2006; Qin et al., 2009). In addition, significance of Ca^2+^, ROS, and NO in the regulation of various processes in pollen-pistil interaction has been extensively studied. Before pollination, ROS highly accumulate in stigma for the protection from damage caused by bacterial or fungal pathogens (Zafra et al., 2010; Suzuki and Katano, 2018). Following attachment of pollen to stigma, NO-ROS redox signaling activates ROS scavenging enzymes derived from pollen coats. After pollen germination, pollen tube elongation is induced by Ca^2+^-dependent activation of RESPIRATORY BURST OXIDASE HOMOLOG H and J in the tip of pollen tube (Kaya et al., 2014). Furthermore, orientation of pollen tube growth to ovule also involves NO and Ca^2+^ signaling (Prado et al., 2008; Traverso et al., 2013). NO as a negative chemoattractant substance accumulates in whole ovary except for micropyle. Ca^2+^ in the tip of pollen tube functions as a key factor that leads to successful fertilization through correct navigation of pollen tubes to micropyle (Prado et al., 2008). In contrast to NO, POLLEN RECEPTOR-LIKE PROTEIN KINASE 6 (PRK6) and RHO OF PLANT GUANINE NUCLEOTIDE EXCHANGE FACTORS (ROPGEFs) function as positive regulators to lead pollen tube into micropyle (Okuda et al., 2009; Takeuchi and Higashiyama, 2016).

Based on these findings together with our results, we hypothesized that crosstalk between Ca^2+^, ROS, and NO might be involved in the modulation of morphology in stigma depending on status of pollen attachment, leading to the maintenance of proper pollen-pistil interaction. Stigma without pollen showed high accumulation of O_2_^-^, while O_2_^-^ accumulation decreased when many pollen attached to the stigma (**Supplementary Figures S9A–D**). Nevertheless, stigma without pollen showed almost no accumulation of Ca^2+^, although stigma with pollen accumulated high level of Ca^2+^ (**Figure 5**). It is an opposite event of the theory, because Ca^2+^ signaling has been considered as inducer of RBOH dependent O_2_^-^ production (Kadota et al., 2015). Such a unique phenomenon that can be observed on stigmas might be essential for pollen-pistil interaction. Decrease in O_2_^-^ accumulation coincident with previous reports might be due to the release of antioxidant substance from pollen coat into stigma induced by NO-ROS redox signals (Traverso et al., 2013; Katano et al., 2018a). In addition, high accumulation of Ca^2+^ in stigma might be associated with the functions of NO derived from stigma, as well as Ca^2+^ derived from pollen. It was reported that NO activated calcium permeable channel (Clementi, 1998; Besson-Bard et al., 2008).

Our results suggest that pollen attachment to stigma is a quite important factor for morphological alteration of stigmatic papillae. However, it is still unclear what are the factors in pollen, which induce morphological alteration of stigma. One possibility is a pollen coat because it is a primal portion which directly attach to the stigmatic papillae. Further studies will be required to elucidate significance of pollen coat in morphological alteration of stigmatic papillae. A previous study demonstrated that high humidity condition induced morphological alteration of stigmatic papillae in flowers *via* regulation of ABA signaling (Takeda et al., 2018). In our studies, enlargement of stigmatic papillae was observed especially in flowering stage under heat stress, suggesting that abiotic stress response mediated by ABA, an important stress response plant hormone could be involved in stigma enlargement. It should be therefore necessary to uncover the hormone signals that govern the stigma enlargement under heat stress in future studies.

## Data Availability Statement

The raw data supporting the conclusions of this article will be made available by the authors, without undue reservation.

## Author Contributions

KK and NS designed the research. KK and TO performed research. KK analyzed data. KK wrote the paper. NS edited the manuscript. All authors contributed to the article and approved the submitted version.

## Funding

This research is supported by the funding from Sophia University, Tokyo, Japan.

## Conflict of Interest

The authors declare that the research was conducted in the absence of any commercial or financial relationships that could be construed as a potential conflict of interest.
