# Supplementary figures and images for: Failure of Pollen Attachment to the Stigma Triggers Elongation of Stigmatic Papillae in Arabidopsis thaliana
Source: Front Plant Sci. 2020 Jun 30;11:989. doi: 10.3389/fpls.2020.00989 (PMC7340091; doi:10.3389/fpls.2020.00989)

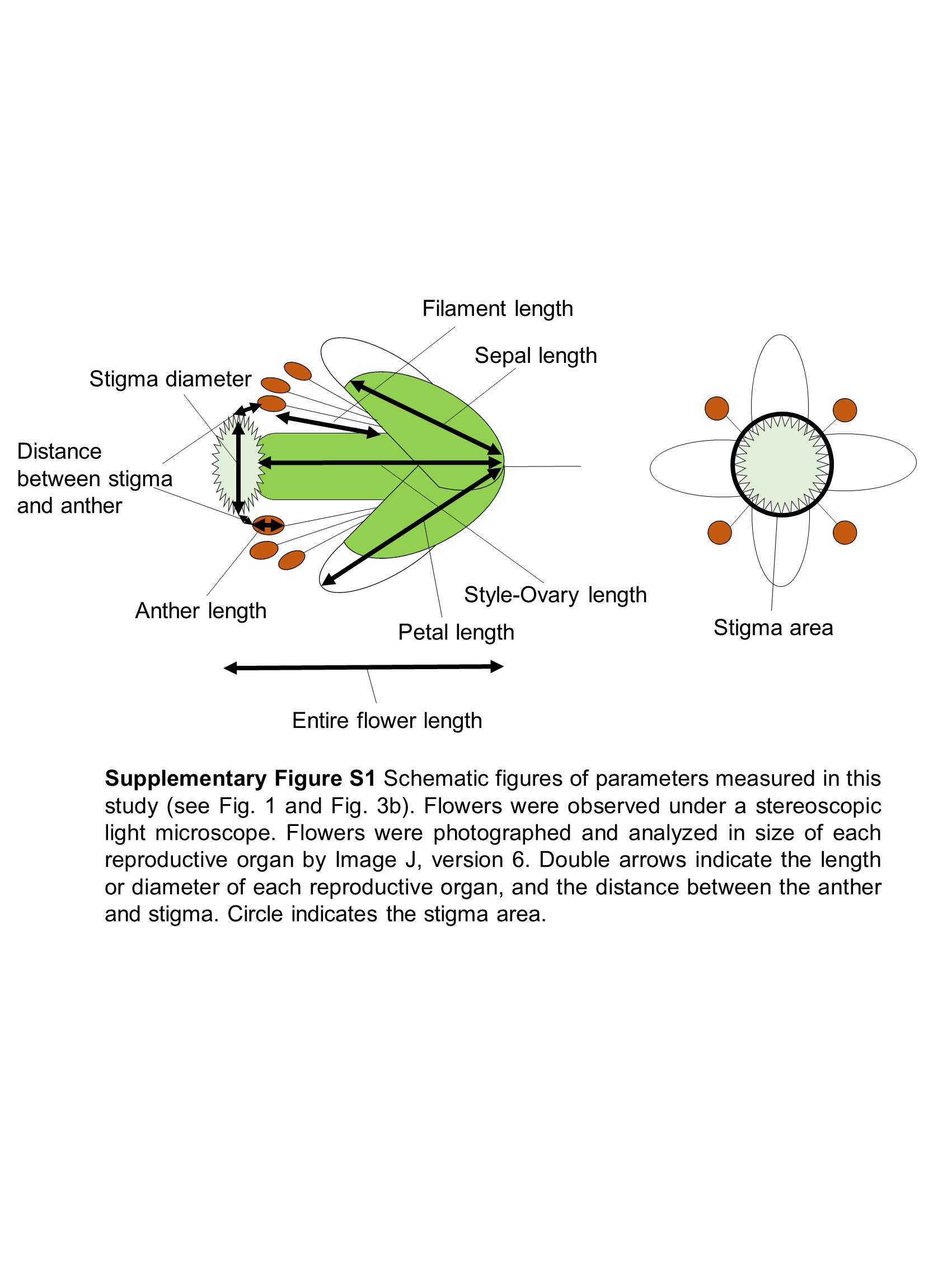

Supplement: Supplementary file 1 [file Image_1.tif]

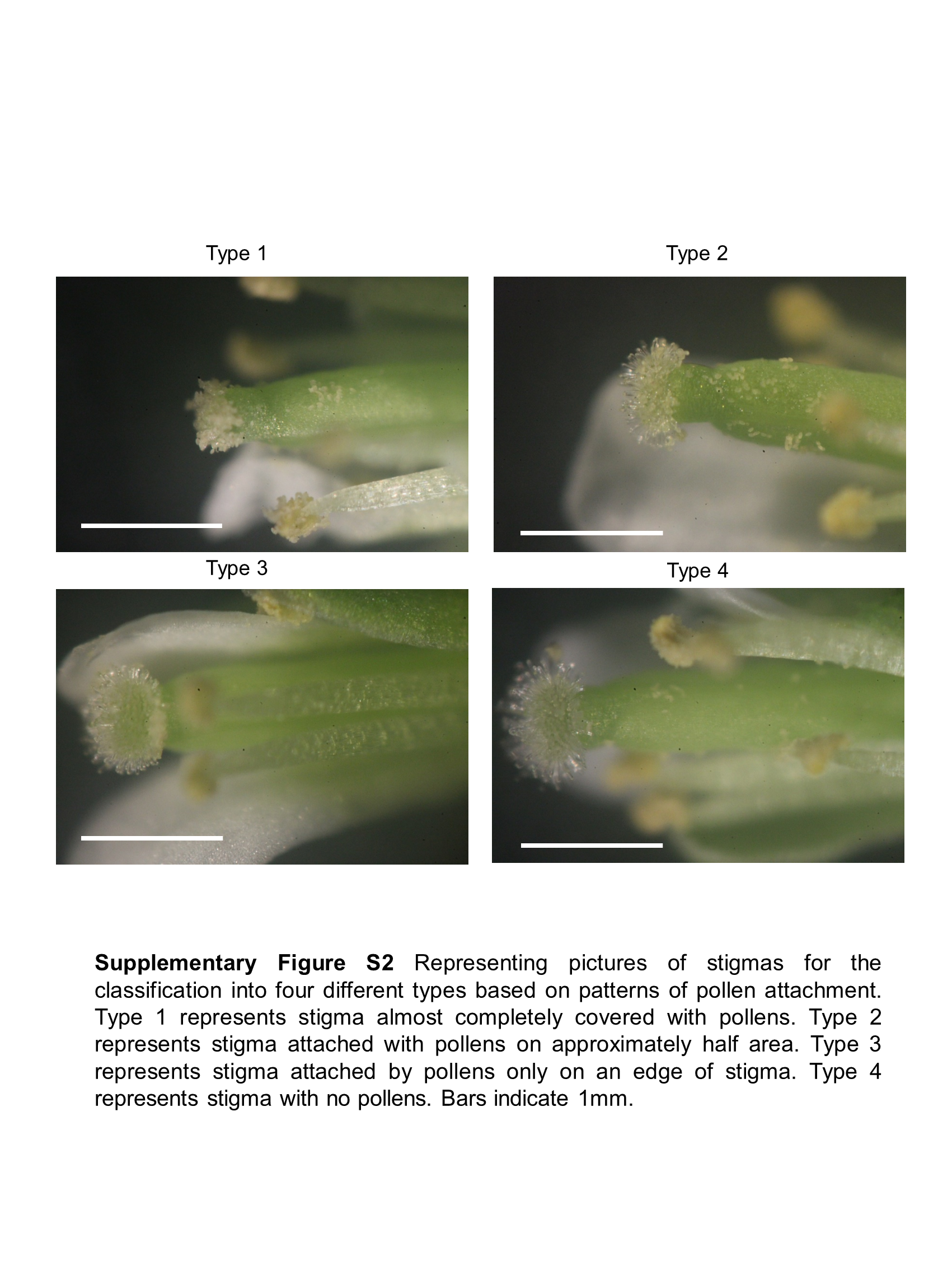

Supplement: Supplementary file 2 [file Image_2.tif]

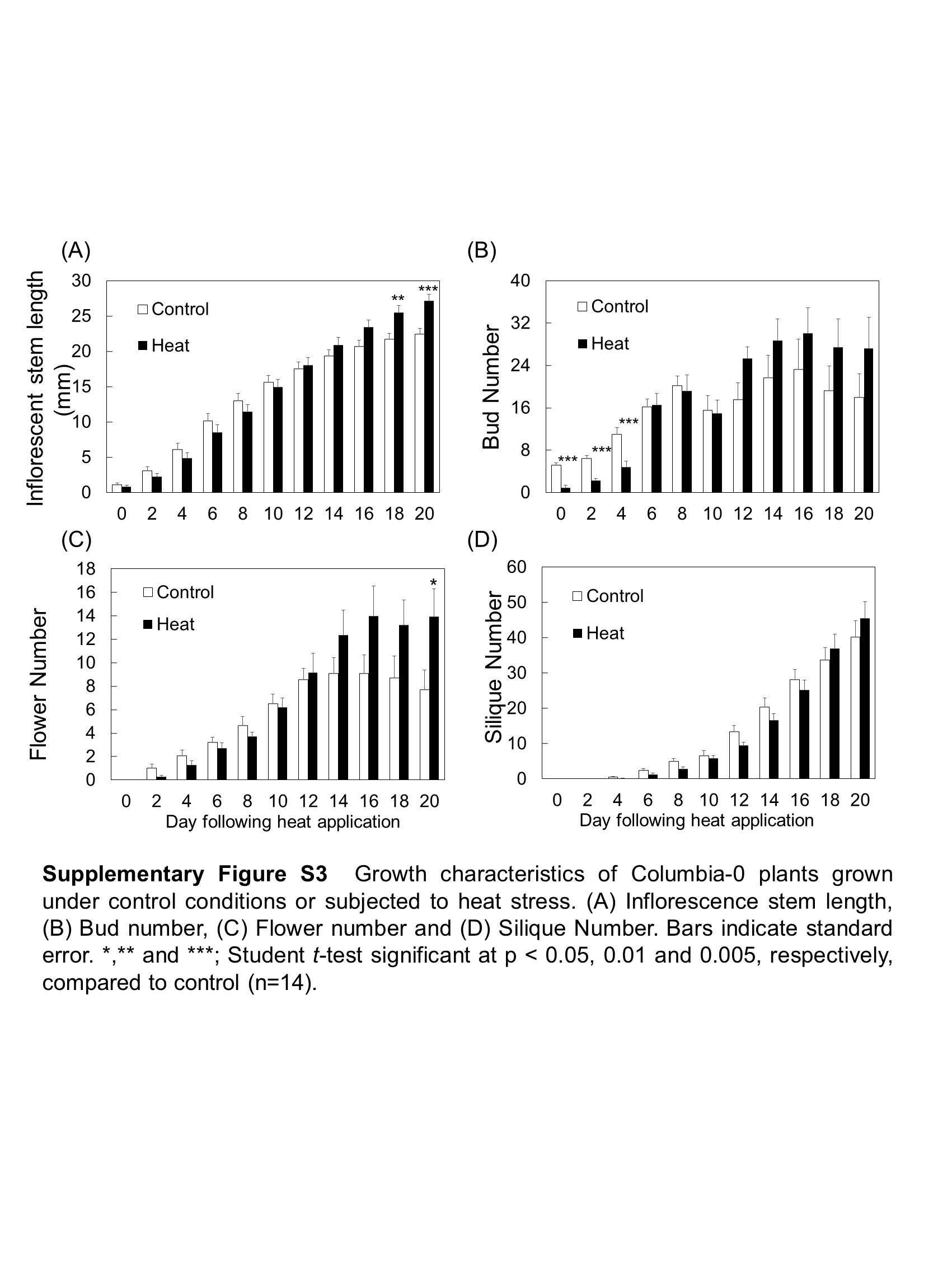

Supplement: Supplementary file 3 [file Image_3.tif]

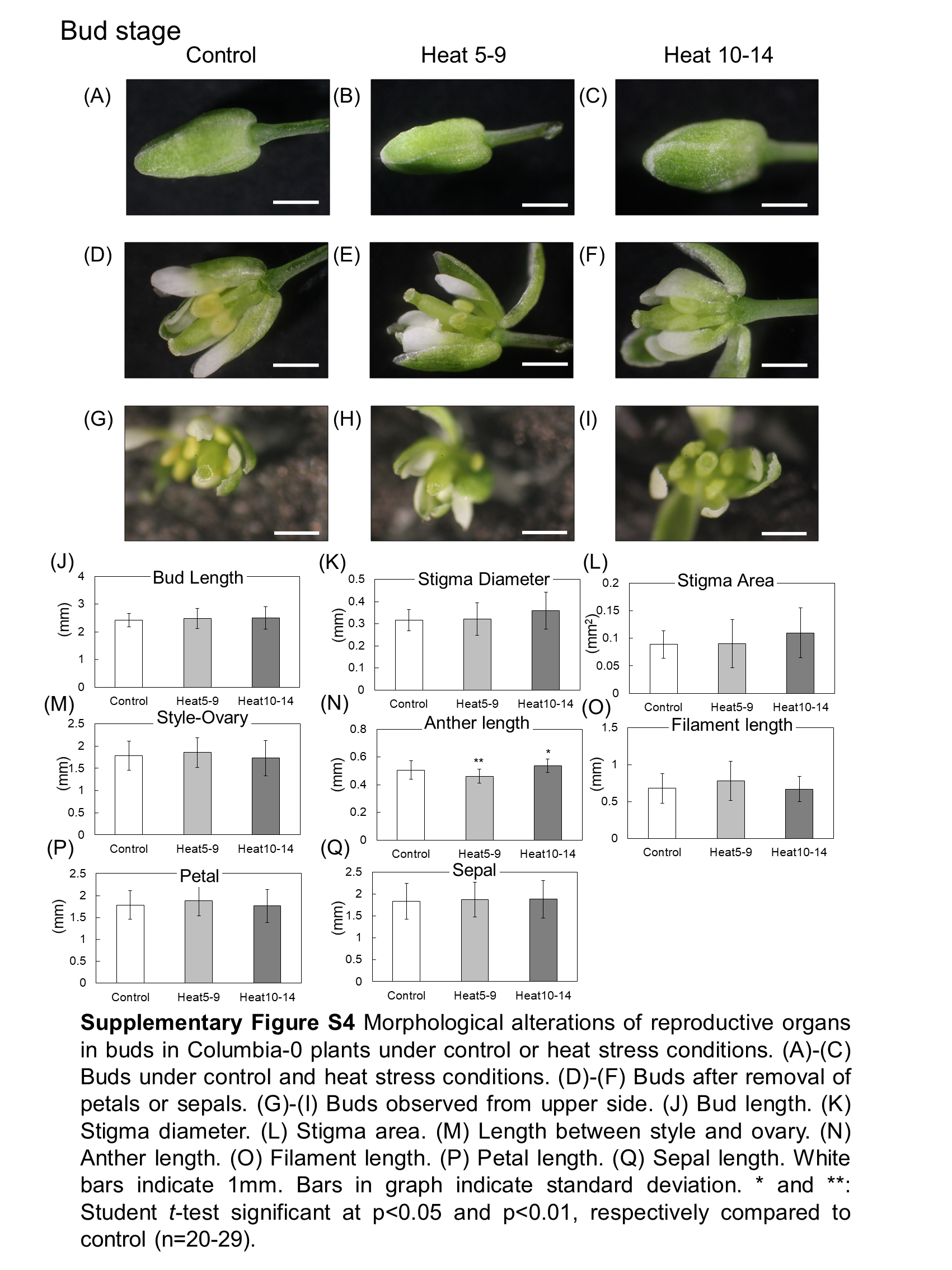

Supplement: Supplementary file 4 [file Image_4.tif]

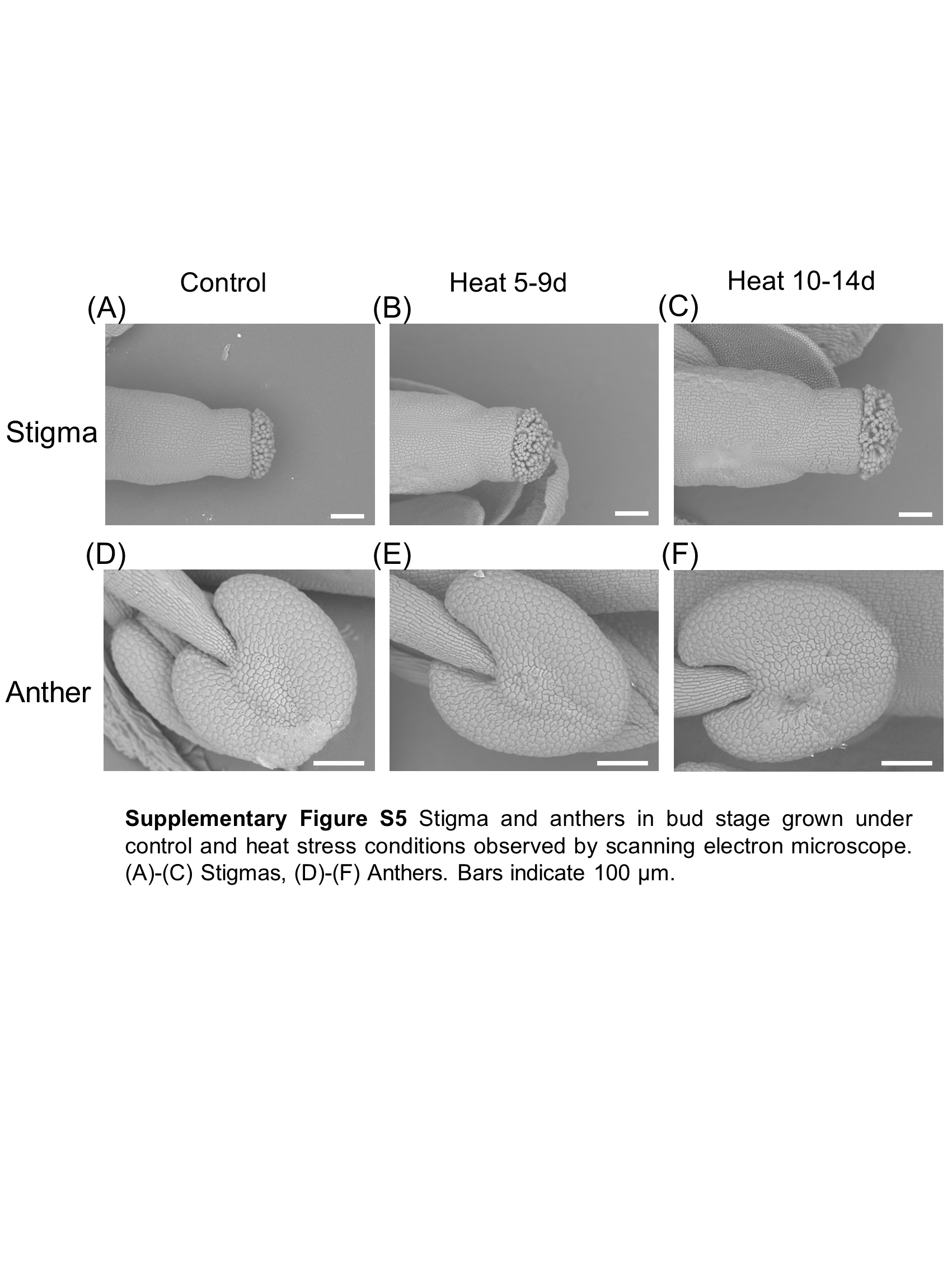

Supplement: Supplementary file 5 [file Image_5.tif]

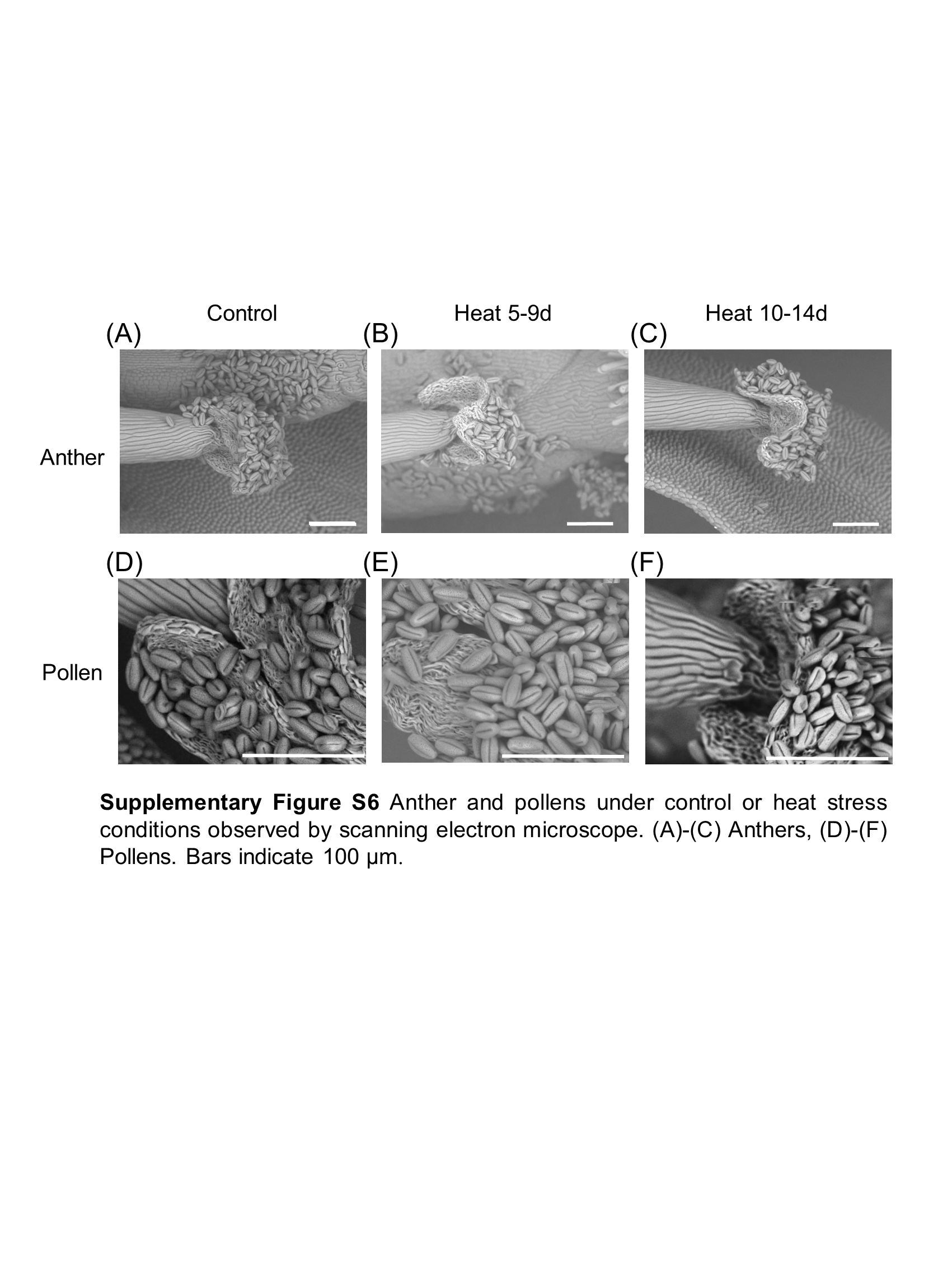

Supplement: Supplementary file 6 [file Image_6.tif]

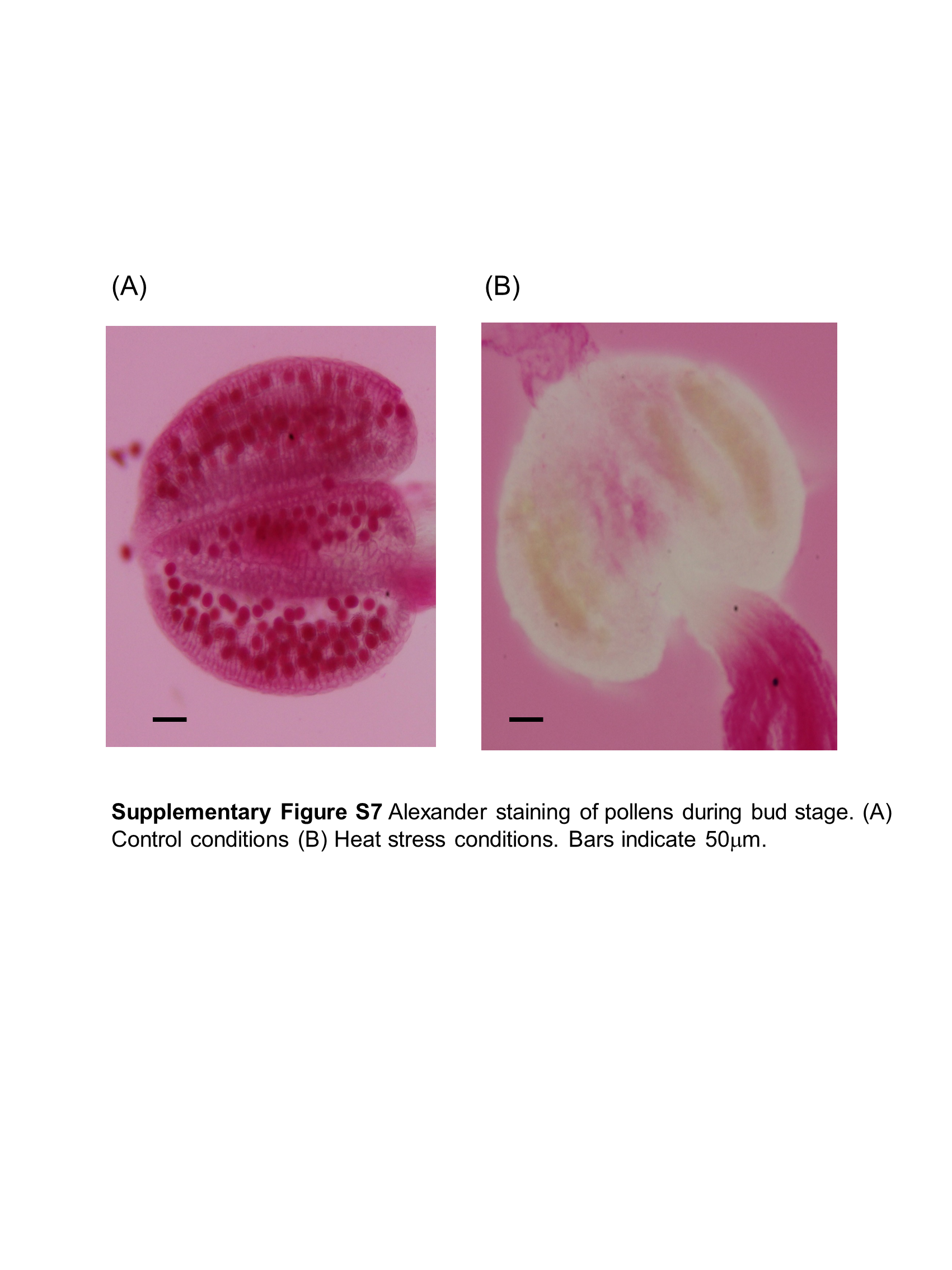

Supplement: Supplementary file 7 [file Image_7.tif]

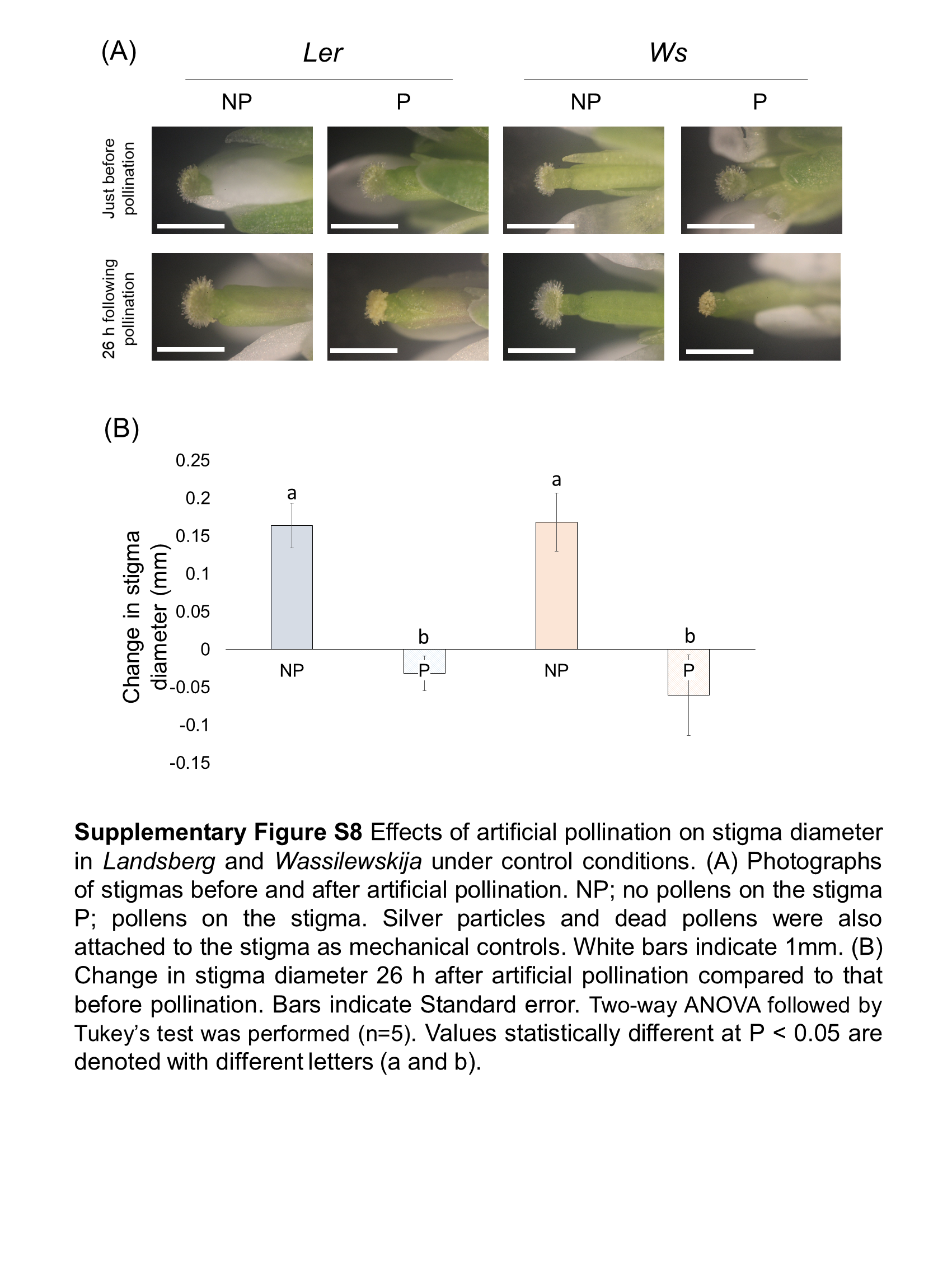

Supplement: Supplementary file 8 [file Image_8.tif]

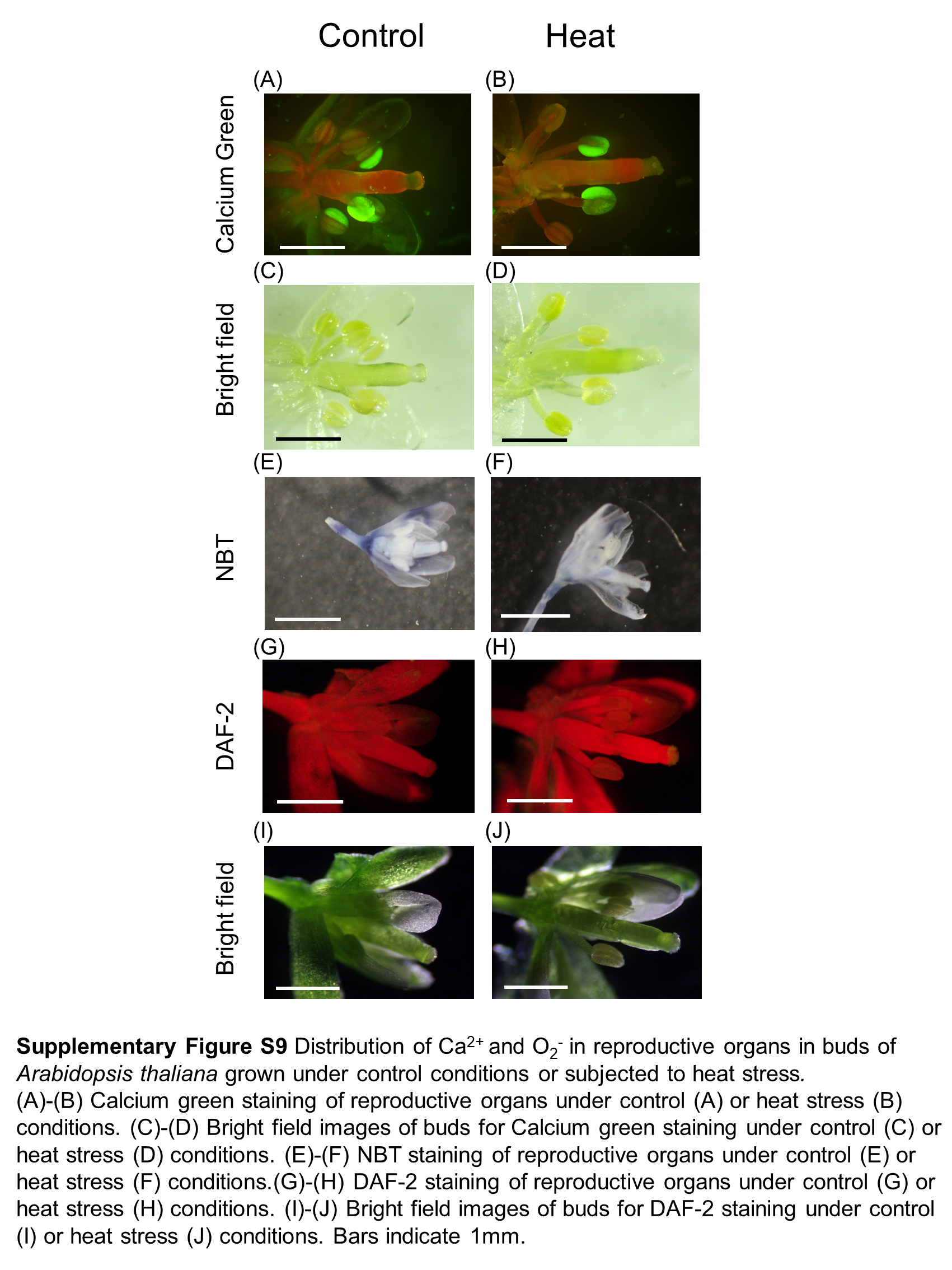

Supplement: Supplementary file 9 [file Image_9.tif]
